# Low fungal knowledge and limited identification skills: study reveals a species literacy gap among laypeople from Germany

**DOI:** 10.1038/s41598-026-41150-w

**Published:** 2026-02-24

**Authors:** Ina Schanz, Martin Remmele

**Affiliations:** Biologie und ihre Didaktik, Trier University, 54296 Trier, Germany

**Keywords:** Biodiversity awareness, Fungal species literacy, Species identification, Species knowledge, Fungal awareness disparity syndrome (FADS), Knowledge gap, Ecology, Ecology, Plant sciences

## Abstract

Biodiversity is one of nature’s greatest assets and is essential for planetary health and human well-being. However, global biodiversity continues to decline at an alarming rate, partly due to limited public engagement in conservation efforts. Previous research has revealed low levels of species knowledge among the public, particularly regarding native plants and animals. However, studies on species literacy with a focus on fungi, one of the most species-rich kingdoms, remain scarce. This study assessed fungal species literacy among a representative sample of laypeople from Germany (*n* = 747) through an identification and knowledge test and examined potential predictors. Identification skills were associated with collecting behavior, rural residence, nature connectedness, and age. On average, participants identified only 16.7% of native fungal species, and more than a quarter (26%) could not identify any. Edibility was correctly assessed in 36.0% of the cases, and the majority believed that fungi are plants. These findings reveal a substantial gap in fungal species literacy, likely perpetuating the continued neglect of fungi in research, conservation and education, which in turn can have negative effects on global conservation goals. To address this gap, the results could provide insights for strengthening fungal representation in school curricula and public education.

## Introduction

Global biodiversity is declining at an unprecedented rate in human history^1^, undermining ecosystem resilience and increasing the risk of instability or collapse faster than predicted^2,3^. This trend threatens the United Nations Sustainable Development Goal (SDG) 15, “Life on Land”, which emphasizes the protection, restoration, and sustainable use of terrestrial ecosystems. Each species plays an indispensable role in ecosystem functioning, forming part of an intricate web. Species themselves constitute the foundation of Earth’s biodiversity, whereas ecosystems serve to sustain and maximize it^4^. Currently, an estimated 47,187^58^ species are considered threatened. Since the ecological impact of a single species is difficult to assess^6^, as ecosystems function as more than the sum of their individual components, the ultimate consequences of biodiversity loss remain uncertain. Although public awareness of biodiversity decline has increased^7^, through international and national conservation initiatives, human activities continue to endanger species persistence^8^. Consequently, one of the key objectives of scientific research^9^ is to identify and address the underlying causes, particularly those stemming from human behaviour^10^.

The multifaceted reasons include a growing disconnection from nature^11,12^, a decline in taxonomic expertise^13^ leading to an identification crisis even among specialists^14^, and insufficient ecological knowledge^15^. Since people tend to value and protect what they know^16^, species literacy is indispensable for effectively maintaining biodiversity^17–19^. The term “species literacy” extends beyond the mere naming of organisms and encompasses both in-depth and broad taxonomic knowledge^19^. Being species-literate entails the ability to identify, name, and understand a species’ behavior, habitat, ecological role, and threats. It also includes observation skills and the ability to apply biological knowledge^19^. Identification skills play a key role in species literacy, which further involves a basic understanding of the species concept itself^20^. Knowledge of species and their life conditions fosters interest in nature and promotes understanding of environmental issues, such as the role of species in ecosystem functioning^21^. Such knowledge encourages more respectful attitudes toward other organisms and greater appreciation of their intrinsic value^22,23^. In turn, it can strengthen emotional connections to nature^24^ and promote positive attitudes^25^. As a result, public engagement can be enhanced that directly or indirectly benefits the natural environment and its associated wildlife based on well-informed decisions^21,26^. These behavioral changes—such as participation in conservation activities, recycling, energy conservation, and environmentally responsible consumption^26^—are crucial for effective conservation. Broad public support is essential, as conservation efforts depend on sustained funding, participation, and social acceptance^27^. A widely shared societal commitment to biodiversity conservation can motivate policymakers to adopt protective measures, whereas limited public concern reduces the likelihood of such action^28^. Hence, species literacy is considered a key predictor of pro-environmental behavior and nature conservation^29–31^, aligning with the United Nations SDG 15.

Despite the critical role of species literacy, international studies have revealed a pronounced lack of species knowledge among the general public. Most research in this field, however, has focused on animals and plants, often highlighting the disparity in attention between these groups. Species knowledge among the general public is generally low^19,32–34^, although it tends to be somewhat greater for animals than for plants^35^. The phenomenon whereby people frequently fail to notice plants in their surroundings, consider them inferior to animals, and underestimate their ecological roles is termed “Plant Awareness Disparity” (PAD). However, species literacy concerning fungi remains poorly studied despite their ecological importance.

Fungi represent one of the most diverse, intricate, and ecologically vital groups of organisms. Their distinctive morphological, physiological, and nutritional traits make them unique among all living organisms^36,37^, and they account for approximately 20% of all eukaryotic species^8,38^. Although primarily terrestrial, fungi inhabit nearly every type of substrate^39^. As the principal decomposers of biomass, they play a central role in both abiotic and biotic cycles and interact with nearly all other life forms—from enabling plant terrestrialization^40^ to farming soil bacteria^41^. For humans, fungi provide essential compounds, including antibiotics and cholesterol-lowering drugs. Given that fungi underpin nearly all terrestrial ecosystems and support the functioning of the biosphere^42^, their diversity is indispensable for global ecosystem stability and resilience.

The few international studies that exist indicate low levels of fungal species literacy^43^ and a broader lack of public awareness, recently described as “Fungal Awareness Disparity Syndrome” (FADS)^41^. FADS encompasses general disinterest in fungi, their association with food, decay, or disease, the inability to recognize fungal presence or activity, limited understanding of fungal ecology, and minimal engagement with fungal conservation^41^. This phenomenon reflects a historical neglect in ecological research^42,44^, resulting in limited baseline data^42^ and substantial knowledge gaps^45^. Fungal distributions are also challenging to document, as many species are small, cryptic, and difficult to identify, often possessing complex and unfamiliar nomenclature^38^. Consequently, fungi continue to be overlooked and overshadowed by plants and animals^38^ in terms of biodiversity monitoring, and fungal conservation^46^. On the IUCN Red List 18% of known plant species and 80% of vertebrate species have been assessed for extinction risk^38^ but only 1300 fungi species (≈ 6% of the total 155,000 described and ≈ 0.8% of the estimated 2.5 million species^3^. Of the fungi species assessed, 411 are currently considered threatened^5^, but their underrepresentation on the Red List suggests that the actual number of endangered species is likely much higher. Fungi face direct threats from deforestation, agricultural intensification, pollution, and climate change, all of which disrupt the delicate environmental conditions required for their growth and reproduction^5^. A striking example is the Chilean bolete (Butyriboletus loyo), now highly endangered due to overharvesting—often of immature specimens and including their mycelium^5^—highlighting a gap in public species literacy. In particular, both aerial and soil fungal communities in human-populated areas are less substantially diverse than in natural habitats^47^.

To adopt a holistic approach to mitigating biodiversity loss^19^, it is essential to enhance fungal species literacy. Accordingly, this study assessed the level of species literacy regarding fungi by examining the familiarity of the population in Germany with native fungal species.

(Q1) How familiar is the population in Germany with common local fungi?

Furthermore, we investigated potential variables related to fungal species literacy to derive scientific, educational and conservation implications. Prior research has shown that species literacy tends to increase with age^16,34,48^ and higher educational attainment^16,19^. Evidence regarding gender effects is mixed: some studies have reported greater performance among males, possibly linked to hunting or outdoor activities^49^, whereas others have reported greater performance among females^50^ or even no significant gender differences^19^. Rural upbringing led to slightly better species literacy^33,51^, although other studies reported no or even inverse effects^19,32,33,50^. Identifying primary sources of species knowledge is also crucial for educational implications. Family environments are frequently cited as the most common source of biodiversity knowledge^33,48,52,53^. Since individuals’ connectedness with nature influences interest development and knowledge acquisition^54^, it may also affect fungal species literacy. Additional predictors could include time spent in nature and mushroom collecting behavior.

(Q2) Which variables are related to fungal species literacy in laypeople from Germany?

The first section presents the results, followed by a discussion and conclusions, and finally, the study methods are clarified.

## Results

The connectedness to nature, measured on a scale from 1 to 7, averaged M_connectedness_ = 4.34 (SD = 1.63), with participants spending on average M_time_ = 9.75 h per week in nature, SD = 12.0. Regarding consumption habits, a large majority (89.2%) reported eating mushrooms. With respect to collecting behavior, 45% of the respondents stated that they had already collected mushrooms, whereas 55% had never done so. Among those not collecting, the most common reasons were a lack of knowledge (34%), lack of interest (15%), other reasons (3%), and local circumstances (3%). Family and friends were identified as the main sources of fungal knowledge, followed by self-directed learning. A total of 112 participants indicated having no knowledge of fungi (Table 1). Only 29% of the participants recalled having covered fungi as a topic in school; 34% stated that fungi had not been addressed in an educational context, and 37% were unsure.

**Table 1:** Sources of knowledge about fungi. The participants indicated their sources of fungal knowledge acquisition in a multiple-choice question (multiple responses possible). The three most frequently cited sources are highlighted in bold.

Source	Participants (%)
Friends/family	**38.9**
Autodidactic (e.g. books)	**33.2**
Preschool/school/university	**27.9**
No existing knowledge	21.1
Other sources	14.8
Leisure activities (e.g. scouts)	9.3

### (Q1) Familiarity with Common Local Fungi

**Fig. 1:**
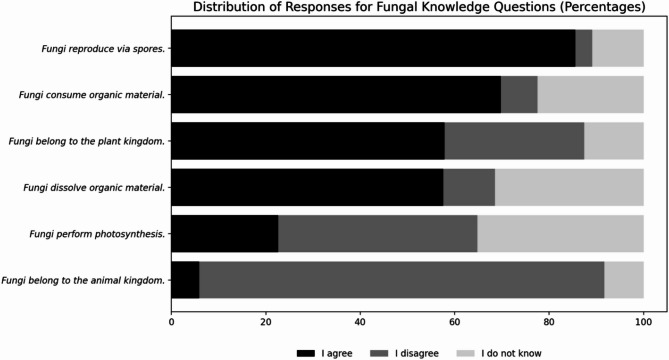
Proportional distribution of participants’ responses to general statements about fungi. The participants either chose between “I agree” (black), “I disagree” (dark gray), or “I do not know” (light gray). Statements are ordered by the proportion of agreement.

The majority of participants (70%) were unable to list five fungal species at random (M_random_=3.09 out of five), and 10% could not name a single species. The most frequently mentioned species were penny bun (*Boletus edulis*), chanterelle (*Cantharellus cibarius)*, champignon mushroom (*Agaricus* sp.), fly agaric (*Amanita muscaria*), and bay bolete (*Imleria badia)*. Together, the participants mentioned over 80 fungal species, including uncommon and habitat-specific taxa such as the golden larch bolete *(Suillus grevillea)* and nonnative species such as enoki (*Flammulina velutipes).* The investigation of the participants’ knowledge (as shown in Fig. 1) revealed that a majority consented that fungi reproduce via spores (86%), consume organic material (70%), are classified with the biological kingdom of plants (58%) and dissolve organic material (58%). In total, a minority agreed that fungi perform photosynthesis (23%) or believed that fungi are animals (6%).

**Fig. 2:**
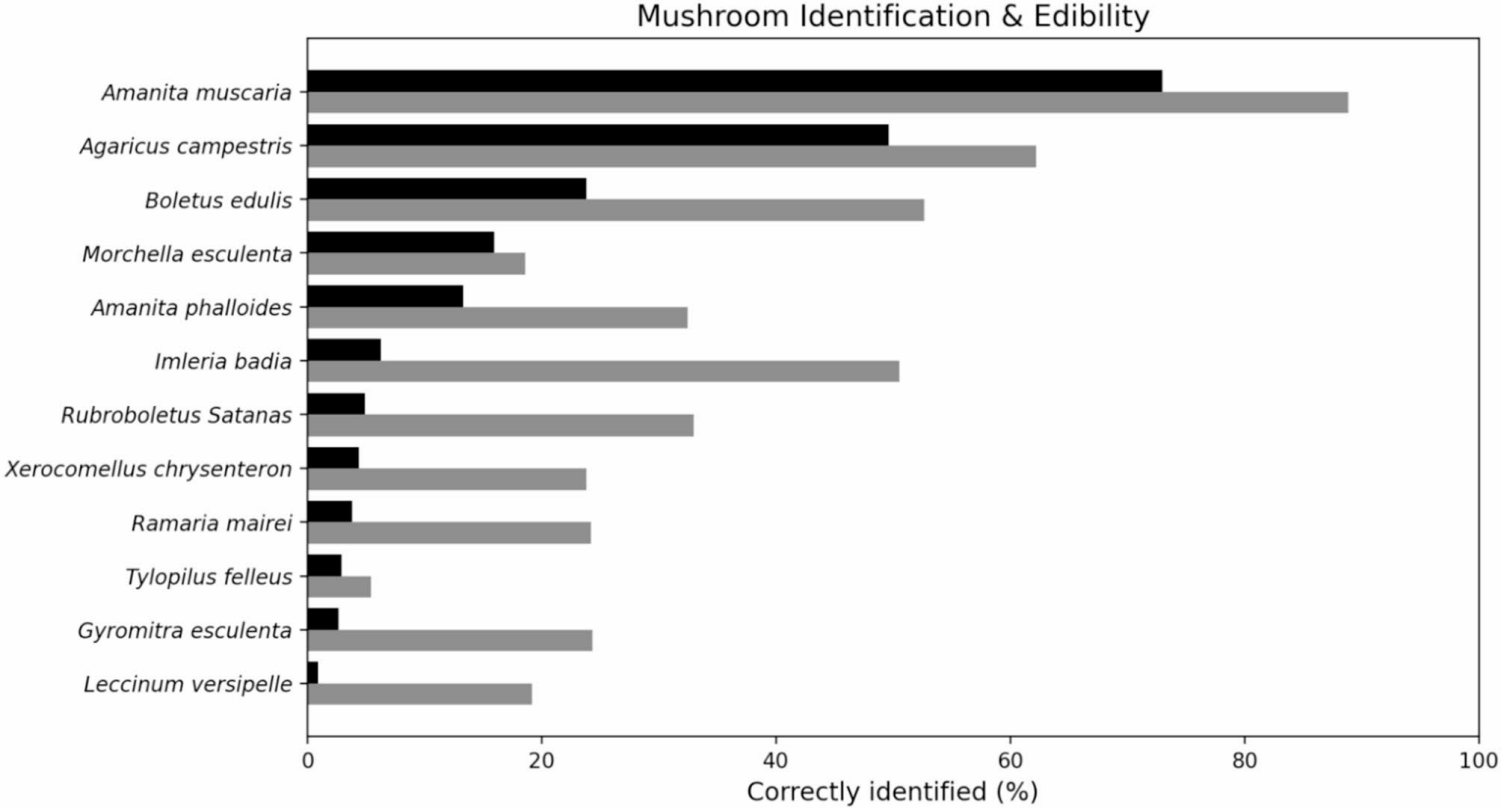
Proportion of laypeople correctly identifying each fungal species and assessing edibility. The black bars indicate identification accuracy; the gray bars represent correct edibility judgments. Species are ordered by an identification rate.

As shown in Fig. 2, participants correctly identified an average of 16.7% of the local fungal species (M_fungi_ = 2.0 out of 12, SD 1.75) and correctly assessed the edibility of 36% (M_edibility_ = 3.0 out of 12). The highest identification rates were achieved for fly agaric mushrooms (*Amanita muscaria)*, field mushrooms (*Agaricus campestris*), and penny buns (*Boletus edulis*). Overall, 26% of the participants were unable to identify a single fungus.

**Fig. 3:**
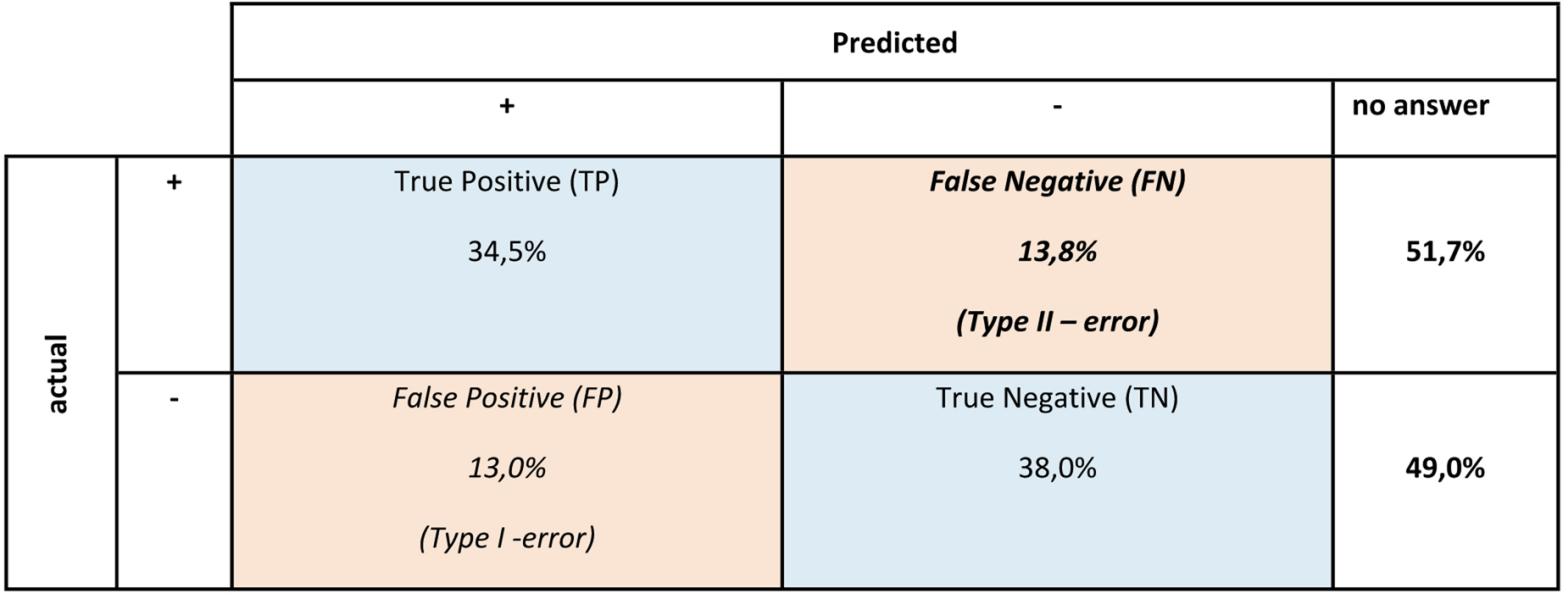
Extended confusion matrix for edibility classification (toxic/inedible = +, edible = -, no answer). The matrix displays the percentage of true positives (TPs), false negatives (FNs; Type II error), false positives (FPs; Type I error), and true negatives (TNs).

In comparison with identification performance, participants generally scored higher in judging food value. To evaluate the accuracy of the edibility assessment, all the responses were analyzed via an extended confusion matrix (Fig. 3). For the answered cases, the model achieved an overall accuracy of 72.8%, quantifying the ratio of correct predictions (true positives + true negatives) to total predictions. The F_1_-score was 72.0%, combining precision and recall into a single metric balancing false positives and false negatives. The sensitivity (correct detection of toxic mushrooms) was 71.4%, the specificity (correct identification of edible mushrooms) was 74.5%, the precision of the toxicity classification was 72.6%, and the negative predictive value was 73.3%. Across all the fungal species presented, 65.5% of the participants either misjudged or were unsure about edibility. Among the edible species, 62.4% of the participants were indecisive or incorrect. Few participants misjudged the field mushroom (*Agaricus campestris)*, whereas most were uncertain or incorrect regarding the red cracking bolete (*Xerocomellus chrysenteron*), the orange birch bolete (*Leccinum versipelle*), and the common morel (*Morchella esculenta*). For inedible but nontoxic species, such as the satan’s bolete (*Rubroboletus Satanas)*, 67.3% of the participants misclassified or were uncertain about edibility. The bitter bolete (*Tylopilus felleus*), which is often considered mildly toxic because it causes stomach upset when raw, was incorrectly judged or unknown by 94.6% of the participants. Among the toxic species, 11.2% misjudged or were unsure about fly agaric (*Amanita muscaria)*, whereas higher misclassification rates were found for the death cap (*Amanita phalloides*, 67.9%), the brain mushroom (*Gyromitra esculenta*, 76.0%), and the coral mushroom (*Ramaria mairei*, 76.0%).

## (Q2) Relationships between variables

A multiple linear regression was conducted to identify predictors of fungal identification skills (dependent count variable). The model was statistically significant, F(5, 741 = 14.52, *p* < .001, R^2^ = 0.089, indicating that the predictors explained 8.9% of the variance in identification skills. The residual variance was 1.284. The significant predictors included mushroom collecting (β = 0.238, *p* < .001), rurality (β = 0.079, *p* = .032), connectedness with nature (β = 0.065, *p* = .012), and age (β = 0.068, *p* = .056). Male gender had a negative association with identification skills (β = − 0.229, *p* = .006). No significant relationships were found for time spent in nature or for diverse gender identities. Because of high multicollinearity (*r* > .35) between age and educational attainment, both variables could not be entered into the model simultaneously; therefore, only age was retained in the final analysis.

A second regression (backward elimination) was carried out, using fungal knowledge as the dependent count variable. The model was statistically significant, F(4, 742) = 7.38, *p* < .001, R^2^ = 0.038, explaining 3.8% of the variance. The significant predictors were collecting behavior (β = 0.13, *p* < .001), connectedness with nature (β = 0.082, *p* = .026), male gender (β = 0.081, *p* = .26), and age (β = 0.080, *p* = .30). The residual variance was 0.386. No significant relationships were observed for weekly time spent in nature or place of residence. Diverse gender identities were excluded because of an insufficient sample size (*n* = 1).

**Fig. 4:**
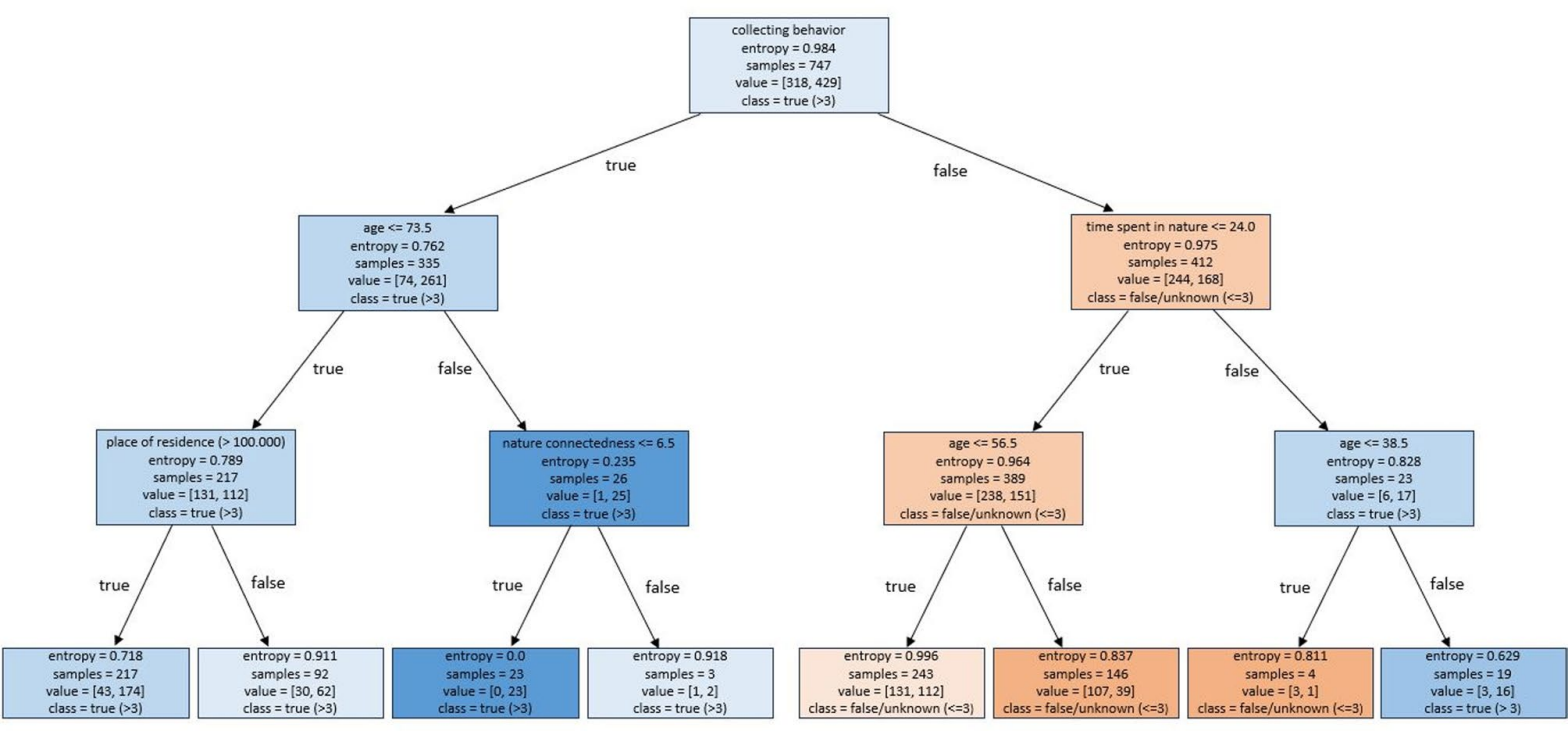
Decision tree for prediction of edibility classification. The tree is read from top to bottom showing the top-most box as the root node, which represents all the people (*n* = 747) in the dataset. Each box from the first three rows gives five pieces of information, i.e. decision rule (top line) which is a question about one of the features (if the answer to the question is “True”, you follow the branch to the left, otherwise to the right); entropy that measures the randomness or impurity of the group of samples in that node; the samples that show the number of people from the dataset are in that particular node/group; the value which shows the distribution of people within that node according to the two possible outcomes [X, Y] while X is the number of people who identified 3 or fewer species correctly and Y is the number of people who identified more than 3 species correctly; class which tells the major outcome for the samples in that node (either ‘true’ or ‘false unknown’). The color of the node reflects the majority class, with darker shades indicating higher purity.

To identify key predictors underlying participants’ ability assess mushroom edibility, a decision tree model was constructed based on the confusion matrix outcomes as shown in Fig. 4. On average, participants correctly identified the edibility of 3 out of 12 fungal species. Therefore, the model classified outcomes as either ‘false/unknown’ (≤ 3 species identified correctly regarding their edibility) or ‘true’ (> 3 species identified in terms of edibility). Participants’ collecting behavior emerged as the strongest predictor and was placed at the root node of the tree. Among participants with prior collecting experience, age constituted the second most influential factor. For individuals aged ≤ 73.5 years, place of residence served as the next most important variable, indicating that living in a large city had a slightly positive impact edibility judgement (*n* = 217, value [43, 174], entropy = 0.7). In contrast, for participants aged ≥ 74, nature connectedness below 6.5 was associated with a higher accuracy in edibility assessment (*n* = 23, value [0, 23], entropy 0.0). This sequence represents the model’s “favorable pathway”, indicating an optimal combination of predictors for correct classification. For participants without collecting experience, time spent in nature per week was the second most important predictor. Those spending ≤ 24 h per week outdoors exhibited a higher likelihood of incorrect edibility assessments. Age emerged as the third most relevant factor in this subgroup: individuals aged ≤ 56.5 years demonstrated a lower risk of misclassification (*n* = 243, value [131, 112], entropy 0.9). Older participants years (*n* = 146, value [107, 39], entropy 0.8) were more likely to predict edibility incorrectly relative to the true labels (false negatives, false positives), following the tree’s “unfavorable pathway”. The misclassification patterns observed in the confusion matrix were reflected in the structure of the decision tree. The unfavorable pathway—characterized by participants without collecting experience, limited time spent in nature, and higher age—accounted for the majority of false-positive and false-negative classifications. Participants following this pathway were more likely to either overestimate the edibility of inedible species (false positives) or underestimate the edibility of safe species (false negatives), consistent with reduced familiarity and lower environmental engagement. In contrast, the favorable pathway, defined by collecting experience, younger age, and greater nature connectedness, corresponded to the lowest entropy values and the highest proportion of correct classifications (true positives and true negatives). These findings suggest that experiential and psychological factors not only determine overall accuracy but also systematically bias the direction of classification errors.

## Discussion

Despite the recognized importance of species literacy in mitigating biodiversity loss, this study revealed a considerable gap in fungal species literacy across multiple dimensions. Notably, participants in this study among laypeople from Germany exhibited low identification skills, highlighting a widespread fungi identification crisis. On average, laypeople identified only 16.7% of the native fungal species, reflecting poor species literacy. The actual identification performance was even lower when considering that both species level and genus level answers were accepted as correct. However, even higher identification accuracy might have been achieved if the participants had been presented real, three-dimensional specimens rather than two-dimensional images. These findings confirm previous studies on species literacy in fungi, animals, and plants^33,50^. The average accuracy of the edibility assessment (36.0%) further points to a substantial knowledge gap. Particularly concerning was the low sensitivity for detecting toxic mushrooms: 13.8% of participants committed type II errors, misclassifying toxic or inedible species as edible. The participants often made systematic misjudgments when encountering dull-colored or inconspicuous fungi, which they tended to classify as edible. For example, the inedible bitter bolete (*Tylopilus felleus*) was regarded as edible by 94% of the respondents. This tendency to classify nonvividly colored mushrooms with edibility could lead individuals to collect and eat them as well. Even the highly toxic death cap (*Amanita phalloides*) was assessed as edible by two-thirds of the participants, and some even misidentified it as a field mushroom (*Agaricus campestris)*. Given that 45% of the respondents had already collected mushrooms, this finding is alarming and potentially life-threatening. Conversely, edible species displaying signal colors were often incorrectly considered toxic. This suggests that participants relied on instinctive color clues rather than factual knowledge when judging edibility. However, toxic mushrooms do not consistently exhibit warning coloration; rather, their unpalatability is more often indicated by distinctive odors^55^. One possible reason is that many mammalian fungivores forage nocturnally and therefore rely less on color vision. A further gap was evident in the participants’ general fungal knowledge. More than two-thirds could not recall whether fungi had been taught in school career or stated that the topic had never been thematized. These results align with previous findings^56^. As in studies on plants and animals^34,48,52,53^, the main source of fungal knowledge was informal learning within private contexts. The participants most frequently demonstrated accurate knowledge concerning fungal reproduction via spores and heterotrophic nutrition through decomposition. However, most failed to recognize fungi as an independent kingdom, often classifying them as plants or animals. Such misconceptions point to a serious taxonomic knowledge gap and a limited understanding of basic biology. Although only 20% believed fungi perform photosynthesis, this result also suggests that photosynthetic activity is not explicitly associated with plants. Every tenth participant was not able to name five fungal species, further demonstrating limited familiarity. The findings of this study can be generalized for Germany to a certain extent, as the sample comprised 747 laypeople representative of the population in Germany. People in other countries may exhibit larger levels of species literacy, reflecting differences in cultural attitudes toward nature, educational systems, and environmental factors.

The main predictor for fungal identification, fungal knowledge and edibility assessment was collecting behavior, highlighting the importance of direct personal engagement with nature in fostering fungal species literacy. Older age was also a positive predictor, which is consistent with the findings of previous studies on animals and plants^16,34,48,56^. An individual’s connectedness with nature is positively correlated with fungal knowledge^11,54^. Gender effects were mixed: male participants scored higher on the fungal knowledge questions, whereas female participants achieved better identification performance. However, gender had no significant influence on participants’ edibility judgement in this dataset. Rurality did not significantly influence fungal knowledge but was positively related to identification ability, which aligns with previous plant studies^34,51^. Individuals in rural areas are likely to encounter fungi more frequently than those in urban settings are. To ensure educational equity, urban students should be offered field-based learning opportunities, such as mushroom identification excursions. The amount of time spent in nature in general had no significant effect on species literacy, likely because time alone does not necessarily imply nature-oriented intention. However, limited time spent in nature had a negative influence on edibility assessment. Nonetheless, the relatively low average weekly time spent outdoors and moderate connectedness scores suggest gradual alienation from nature^12^, which hinders personal engagement with environmental issues.

The observed gaps in species literacy drivers lead to weaker perceptions of biodiversity and a reduced understanding of environmental change. A restricted view of nature can diminish awareness of conservation needs, fostering the misconception that conservation efforts are relevant only elsewhere. Such knowledge gaps impede identification with ecological concerns, reduce active participation in conservation, and threaten the achievement of multiple UN global goals. A lack of knowledge about fungi and their ecological roles can result in lead to underappreciation and neglect in conservation efforts. Ultimately, this knowledge gap undermines progress toward global biodiversity goals, particularly SDG 15. Broad acceptance of biodiversity preservation measures requires higher levels of species literacy^57^. Given that fungi exert major control over ecosystems and are inextricably linked to many SDGs, enhancing fungal literacy is critical.

Unlike other macroorganisms, a substantial amount of fungal diversity remains undescribed^58^ and poorly characterized even among scientists^42,44,45^, as mycological research has historically been underrepresented. Consequently, limited public knowledge about fungi cannot be attributed solely to a lack of interest but should be viewed within the broader context of incomplete scientific understanding. This research gap constrains the depth of information available for inclusion in basic education. Enhancing the representation of fungi in curricula is therefore a valuable goal, but meaningful progress can only be achieved in parallel with continued scientific advances in mycology.

Effective conservation of biological diversity requires flexible and innovative approaches at multiple levels. Internationally, the implementation of UN biodiversity and climate goals that align with the IUCN’s main overarching strategic plan to effectively conserve biodiversity and nature and address the interconnectedness between nature and society must be promoted. Specifically, the IUCN Red List Strategic Plan (2021–2023) aims to expand biodiversity monitoring by assessing at least 260,000 species; fungi should be more integrated. In German science education, curricula largely focus on animals and plants. When addressed, fungi appear mainly in human biology (as pathogens) or in ecological contexts such as forest ecosystems or lichen symbiosis. Accordingly, fungal knowledge is imparted fragmentarily rather than coherently, hindering students from developing an integrated understanding. Given that fungi constitute the second largest biological kingdom and perform diverse and essential functions, they should be integrated more holistically across multiple educational contexts to prevent misconceptions regarding their global significance. At the national level, countries should enhance mycological education by incorporating fungi into school curricula and teacher training. Chile, for example, has implemented a “spiral mycelial education” framework to promote fungal awareness and knowledge^41^. This approach integrates topics such as classification, taxonomy, functionality, morphology, interdependence, and anthropogenic impacts. In addition, Chilean law requires companies and government agencies to include fungi in environmental impact assessments of projects and exemplary measures to ensure fungal conservation. Local and regional initiatives are equally important. To counteract low fungal awareness, outdoor education programmes are essential pathways to experiential learning and engagement. As fungi remain underrepresented in social scientific research, a more fundamental and applied science approach is essential, particularly on their ecological functions and potential roles in food security and ecosystem health. Future comparative studies could examine the existence of Fungal Awareness Disparity Syndrome (FADS), a assessing species literacy for fungi, animals, and plants simultaneously^41^.

## Methods

The present study was based on a survey targeting laypeople from Germany. The quantitative data were collected through an online questionnaire administered via a German online research panel. Prior to data collection, the experimental protocol was approved by a named institutional and licensing committee (Research Ethics Committee of Trier University, Germany; file number EK Nr. 60/2024). The committee confirmed that all research procedures complied with institutional and national ethical guidelines. To ensure compliance with data protection regulations, participation was fully anonymous, and no personally identifiable information was collected. All potential participants received plain-language information on the study’s purpose, procedures, researcher qualifications, and data protection measures. They were also explicitly informed of their right to withdraw from participation at any time without penalty. Informed consent was obtained electronically from all participants prior to inclusion. A pilot test with 15 individuals (not included in the final sample) was conducted to evaluate the questionnaire’s clarity and content validity. No modifications were needed. Data collection took place in September 2024.

A total of 747 participants aged 18–88 years (M = 50.6, SD = 15.9) participated in the study. Participants were recruited via an established pre-existing consumer panel managed by *Consumerfieldwork GmbH* using quota sampling to ensure approximate representation and demographic diversity of the population in Germany. The sample was heterogenous in terms of sex (51% female, 49% male, 0.1% diverse), age (25% 18–34 years, 23% 35–49 years, 27% 50–64 years, 25% 65–99 years), education level based on highest completed qualification (33% lower, 34% intermediate, 33% higher), and place of residence (24% rural area, 22% small town, 25% medium-sized city, 29% large city). Panel members were invited by the consumer panel to participate via email and received a small monetary compensation upon completion of the survey. Participation was voluntary, and all responses were anonymous. Inclusion criteria required participants to be at least 18 years old and residents of Germany. Data quality was further ensured through the inclusion of an attention-check item (a question, where participants had to select a specific answer on a grid) designed to detect careless responding, such as “straightliners”. Respondents failing these checks were flagged and excluded from analyses. Additionally, identity verification and internal quality scores provided by the panel were used to prevent duplicate or fraudulent responses.

The questionnaire comprises two parts. The first part collected sociodemographic data and general knowledge about fungi; the second part assessed native fungal species identification and edibility evaluation. Sociodemographic variables included gender, age, highest educational attainment (9-point scale), and current and longest place of residence (classified as large city > 100,000 inhabitants; medium-sized city 20,000–100,000; small town 5,000–20,000; rural area < 5,000), as they have proven themselves potential drivers of species literacy in previous studies. The participants’ nature connectedness was measured via a single-item seven-point graphical Likert scale based on the Inclusion of Nature in Self measure^59^, which allows the participants to indicate the extent to which they consider themselves part of nature (1 = not at all, 7 = very much). Weekly time spent in nature was also reported. The participants were asked whether fungi had been covered during their school education and to list up to five fungal species familiar to them (native or nonnative). They then responded to general statements about fungi, choosing among “I agree”, “I do not agree”, or “I do not know”. The participants also indicated their sources of knowledge (e.g., family, books, school) or whether they lacked knowledge. Finally, mushroom-eating and mushroom-collecting habits were surveyed, and non-collectors were asked to provide reasons. In the second part, participants’ familiarity with 12 native fungal species was tested via high-quality color images of fruiting bodies (Table 2). The order was randomized to avoid order effects. They were asked to identify each species (e.g., *Agaricus campestris*,* Leccinum versipelle*) and to assess its edibility (edible, inedible/poisonous, or unknown). Species were selected according to the following criteria: (1) native to Germany, (2) commonly encountered, (3) displaying characteristic morphological features (e.g., cap, stipe, veil, color), (4) representing different taxonomic phyla (*Basidiomycota* and *Ascomycota*), and (5) covering both edible and inedible/toxic species. The average completion time was 5.6 min (SD = 2.4 max = 14.1 min).

The raw data were organized and cleaned with Microsoft Excel (v. 2508) and Python (v. 3.11). The variables were encoded and prepared for regression analysis via the libraries *pandas*,* numpy*,* scipy/statmodels*,* matplotlib*, and *seaborn*.

**Table 2:** Fungal species presented for identification.

Common name	Scientific name
Bay bolete	*Imleria badia*
Bitter bolete	*Tylopilus felleus*
Brain mushroom	*Gyromitra esculenta*
Common morel	*Morchella esculenta*
Coral mushroom	*Ramaria mairei*
Death cap	*Amanita phalloides*
Field mushroom	*Agaricus campestris*
Fly agaric	*Amanita muscaria*
Orange birch bolete	*Leccinum versipelle*
Penny bun	*Boletus edulis*
Red cracking bolete	*Xerocomellus chrysenteron*
Satan’s bolete	*Rubroboletus satanas*

Free-text answers were corrected for typographical and autocorrect errors using a two-step phonetic conformity test. First, the Levenshtein distance (implemented via the fuzzy.ratio from *fuzzywuzzy*) was computed to measure string similarity between participant responses and valid species names (score range 0-100). Entries with a similarity score > 80 were accepted as matches (e.g., “peny bun” matched with “penny bun” (*Boletus edulis)* because a close match indicated by a high similarity score of 89). Second, phonetic similarity was verified via the *Soundex* algorithm (*soundex_man* package), which encodes words based on sound, regardless of spelling differences (e.g., “bay bolet” matched with the valid entry “bay bolete” (*Imleria badia))*. Combining both approaches ensures robust matching while minimizing false negatives. An answer was considered correct if the participant named the species or genus (e.g., “champignon” or *Agaricus* accepted for *Agaricus campestris*; *Amanita* accepted for *Amanita muscaria* and *Amanita phalloides).* Identification scores were computed as the number of correctly identified species per participant. Multiple-choice questions assessing general fungal knowledge and edibility were scored automatically. Edibility classification performance was evaluated via an extended confusion matrix including toxic/inedible (+), edible (-), and no answer categories. The matrix displayed true positives (TPs; toxic or inedible species correctly identified), false negatives (FNs; toxic mushrooms misclassified as edible), false positives (FPs; edible species misclassified as toxic), and true negatives (TNs; edible species correctly identified). In order to detect the most relevant predictors of edibility classification, an analysis was performed using a decision tree via the Python library *sklearn.tree* (DecisionTreeClassifier).

To identify drivers of fungal literacy, correlation analyses and multiple linear regression models were applied. Metrical variables (age, time spent in nature) were used directly; ordinal variables (nature connectedness, education level, rurality) were treated as continuous. Collecting behavior and gender were dummy-coded. Two multiple regression analyses were carried out to determine the influence of demographic and behavioral factors on fungal literacy (count variable) and knowledge (count variable). Standard diagnostic tests (t-tests for regression coefficients; F-tests for model fit, variance inflation factor (VIF) and tolerance for multicollinearity) were conducted. All pairwise correlations between the independent variables were less than *r* = .35, indicating no problematic multicollinearity. Backward stepwise selection (*p* > .10 removal threshold) was applied to derive the most parsimonious model and identify the five most significant predictors. Model assumptions were checked visually via Q‒Q plots of residuals. All tests were two-tailed, with the significance level set at α = 5%.

## Data Availability

All data generated or analyzed during this study are included in this published article.

## References

[1] Chapin, F. S. et al. Consequences of changing biodiversity. *Nature* **405**, 234–242. 10.1038/35012241 (2000). 10821284 10.1038/35012241

[2] Keck, F. et al. The global human impact on biodiversity. *Nature*10.1038/s41586-025-08752-2 (2025). 40140566 10.1038/s41586-025-08752-2 PMC12058524

[3] IPBES. Global assessment report on biodiversity and ecosystem services of the Intergovernmental Science-Policy Platform on Biodiversity and Ecosystem Services, (2019).

[4] Gatti, R. C., Hordijk, W. & Kauffman, S. Biodiversity is autocatalytic. *Ecol. Model.* **346**, 70–76. 10.1016/j.ecolmodel.2016.12.003 (2017).

[5] IUCN. IUCN red List of Threatened Species. (2025). Available at https://iucn.org/resources/conservation-tool/iucn-red-list-threatened-species

[6] Hébert, K. Biodiversity change is more than the sum of its parts. *Nat. Rev. Biodivers.*10.1038/s44358-025-00048-7 (2025).

[7] Díaz, S. et al. Summary for policymakers of the global assessment report on biodiversity and ecosystem services of the intergovernmental science-policy platform on biodiversity and ecosystem services. (2019). Available at https://www.ipbes.net/system/files/2021-06/2020%20IPBES%20GLOBAL%20REPORT(FIRST%20PART)_V3_SINGLE.pdf

[8] Taylor, J. W. & Berbee, M. L. Dating divergences in the fungal tree of life: Review and new analyses. *Mycologia* **98**, 838–849. 10.1080/15572536.2006.11832614 (2006). 17486961 10.3852/mycologia.98.6.838

[9] Nature Insight Biodiversity. *Nature* 405, 207; (2000). 10.1038/35012215

[10] O’Brien, K., Garibaldi, L. & Agrawal, A. IPBES Transformative Change Assessment: Full report, (2024).

[11] Nisbet, E. K. & Zelenski, J. M. The NR-6: A new brief measure of nature relatedness. *Front. Psychol.* **4**, 813. 10.3389/fpsyg.2013.00813 (2013). 24198806 10.3389/fpsyg.2013.00813 PMC3814587

[12] DeVille, N. V. et al. Time Spent in Nature Is Associated with Increased Pro-Environmental Attitudes and Behaviors. *Int. J. Environ. Res. Public. Health*. 10.3390/ijerph18147498 (2021). 34299948 10.3390/ijerph18147498 PMC8305895

[13] Páll-Gergely, B. et al. Identification crisis: A fauna-wide estimate of biodiversity expertise shows massive decline in a Central European country. *Biodivers. Conserv.* **33**, 3871–3903. 10.1007/s10531-024-02934-6 (2024).

[14] Paxton, R. et al. Entomology: The bee-all and end-all. *Nature* **521** (9), 57. 10.1038/521S57a (2015). 25992674 10.1038/521S57a

[15] Atran, S., Medin, D. & Ross, N. Evolution and devolution of knowledge: A tale of two biologies. *Royal Anthropol. Inst.* **10**, 395–420. 10.1111/j.1467-9655.2004.00195.x (2004).

[16] Balmford, A., Clegg, L., Coulson, T. & Taylor, J. Why conservationists should heed Pokémon. *Sci. (New York N Y)*. **295**, 2367. 10.1126/science.295.5564.2367b (2002). 11924673 10.1126/science.295.5564.2367b

[17] Wolff, L. A. & Skarstein, T. H. Species learning and biodiversity in early childhood teacher education. *Sustainability* **12**, 3698. 10.3390/su12093698 (2020).

[18] Palmberg, I. et al. Nordic student teachers’ views on the importance of species and species identification. *J. Sci. Teacher Educ.* **29**, 397–419. 10.1080/1046560X.2018.1468167 (2018).

[19] Hooykaas, M. J. et al. Identification skills in biodiversity professionals and laypeople: A gap in species literacy. *Biological Conservation* (2019).

[20] Aldhebiani, A. Y. Species concept and speciation. *Saudi J. Biol. Sci.* **25**, 437–440. 10.1016/j.sjbs.2017.04.013 (2018). 29686507 10.1016/j.sjbs.2017.04.013 PMC5910646

[21] Palmberg, I. et al. Nordic–baltic student teachers’ identification of and interest in plant and animal species: The importance of species identification and biodiversity for sustainable development. *J. Sci. Teacher Educ.* **26**, 549–571. 10.1007/s10972-015-9438-z (2015).

[22] Mohneke, M., Erguvan, F. & Schlüter, K. Explorative study about knowledge of species in the field of early years education. *Journal Emergent Science*, 11–22 (2016).

[23] Remmele, M. & Lindemann-Matthies, P. Dead or alive? teacher students’ perception of invasive alien animal species and attitudes towards their management. *EURASIA J. Math. Sci. Tech. Ed.*10.29333/ejmste/115105 (2020).

[24] Cox, D. T. C. & Gaston, K. J. Likeability of garden birds: Importance of species knowledge & richness in connecting people to nature. *PloS one*. **10**, e0141505. 10.1371/journal.pone.0141505 (2015). 26560968 10.1371/journal.pone.0141505 PMC4641628

[25] Petra L‐M. Loveable’ mammals and ‘lifeless’ plants: How children’s interest in common local organisms can be enhanced through observation of nature. *Int. J. Sci. Educ.* **27**, 655–677. 10.1080/09500690500038116 (2005).

[26] Soga, M. & Gaston, K. J. Do people who experience more nature act more to protect it? A meta-analysis. *Biol. Conserv.* **289**, 110417. 10.1016/j.biocon.2023.110417 (2024).

[27] HOME, R., Keller, C., Nagel, P., Bauer, N. & Hunziker, M. Selection criteria for flagship species by conservation organizations. *Envir Conserv.* **36**, 139–148. 10.1017/S0376892909990051 (2009).

[28] Shwartz, A., Turbé, A., Simon, L. & Julliard, R. Enhancing urban biodiversity and its influence on city-dwellers: An experiment. *Biol. Conserv.* **171**, 82–90. 10.1016/j.biocon.2014.01.009 (2014).

[29] Barron, E. S. Conservation of Abundance. *Conserv. Soc.* **21**, 99–109 (2023). 10.4103/cs.cs_23_22.

[30] Schulte, R. et al. Eine Strategie zur Förderung der Artenkenntnis. *Naturschutz und Landschaftsplanung*. **51** (05), 210–217 (2019).

[31] Silva-Flores, P. et al. Mycorrhizal science outreach: Scope of action and available resources in the face of global change. *Plants People Planet.* **3**, 506–522. 10.1002/ppp3.10213 (2021).

[32] Gerl, T. & Randler, C. Jana Neuhaus, B. Vertebrate species knowledge: An important skill is threatened by extinction. *Int. J. Sci. Educ.* **43**, 928–948. 10.1080/09500693.2021.1892232 (2021).

[33] Remmele, M., Lindemann-Matthies, P. & Like father like son? On the relationship between parents’ and children’s familiarity with species and sources of knowledge. *Eurasia J. Math. Sci. & Technol. Education* **14** (2018).

[34] Enzensberger, P., Schmid, B., Gerl, T. & Zahner, V. Robin Who? Bird species knowledge of german adults. *Anim. Open. Access. J. MDPI*. 10.3390/ani12172213 (2022). 10.3390/ani12172213 PMC9454614 36077931

[35] Remmele, M. & Lindemann-Matthies, P. Beautiful and wanted – how young people perceive invasive alien plant species and certain options of their management. *Int. J. Sci. Educ. Part. B*. **14**, 331–352. 10.1080/21548455.2023.2277706 (2024).

[36] Nagy, L. G. et al. Six key traits of fungi: Their evolutionary origins and genetic bases. *Microbiol. Spectr.* **5**10.1128/microbiolspec.FUNK-0036-2016 (2017). 10.1128/microbiolspec.funk-0036-2016 PMC11687519 28820115

[37] Corbu, V. M., Gheorghe-Barbu, I., Dumbravă, A. Ș., Vrâncianu, C. O. & Șesan, T. E. Current insights in fungal importance: A comprehensive review. *Microorganisms*10.3390/microorganisms11061384 (2023). 37374886 10.3390/microorganisms11061384 PMC10304223

[38] Niskanen, T. et al. Pushing the frontiers of biodiversity research: Unveiling the global diversity, distribution, and conservation of fungi. *Annu. Rev. Environ. Resour.* **48**, 149–176. 10.1146/annurev-environ-112621-090937 (2023).

[39] Peay, K. G., Kennedy, P. G. & Talbot, J. M. Dimensions of biodiversity in the Earth mycobiome. *Nat. Rev. Microbiol.* **14**, 434–447. 10.1038/nrmicro.2016.59 (2016). 27296482 10.1038/nrmicro.2016.59

[40] Talbot, N. J. A cure for ‘fungus blindness’. *Nat. Plants*. **6**, 1068–1069. 10.1038/s41477-020-00767-z (2020).

[41] Waller, A. Mycelial education to cure fungal awareness disparity syndrome. *J. Biol. Educ.* **59**, 391–392. 10.1080/00219266.2025.2493398 (2025).

[42] Desprez-Loustau, M. L. et al. The fungal dimension of biological invasions. *Trends Ecol. Evol.* **22**, 472–480. 10.1016/j.tree.2007.04.005 (2007). 17509727 10.1016/j.tree.2007.04.005

[43] Aigner, L. & Krisai-Grailhuber, I. Eine ethnomykologische Studie über das Pilzwissen in der Bevölkerung des Waldviertels. *Österreichische mykologische Gesellschaft*, 209–224 (2016).

[44] Pautasso, M. Fungal under-representation is (slowly) diminishing in the life sciences. *Fungal Ecol.* **6**, 129–135. 10.1016/j.funeco.2012.04.004 (2013).

[45] Runnel, K. et al. Toward harnessing biodiversity-ecosystem function relationships in fungi. *Trends Ecol. Evol.* **40**, 180–190. 10.1016/j.tree.2024.10.004 (2025). 39532622 10.1016/j.tree.2024.10.004

[46] Drechsler-Santos, E. R. et al. Brazil as a global player in fungal conservation: A rapid shift from neglect to action. *Perspect. Ecol. Conserv.* **23**, 246–254. 10.1016/j.pecon.2025.08.006 (2025).

[47] Abrego, N. et al. Fungal communities decline with urbanization-more in air than in soil. *ISME J.* **14**, 2806–2815. 10.1038/s41396-020-0732-1 (2020). 32759974 10.1038/s41396-020-0732-1 PMC7784924

[48] Jaun-Holderegger, B. *Wege zur Artenkenntnis: eine Untersuchung mit Schülerinnen und Schülern der Mittelstufe im Kanton Bern, Schweiz.* (2019).

[49] Nyhus, P. J., Tilson, R. & Sumianto & Wildlife knowledge among migrants in southern Sumatra, Indonesia: Implications for conservation. *Envir Conserv.* **30**, 192–199. 10.1017/S0376892903000183 (2003).

[50] Gerl, T., Almer, J., Zahner, V. & Neuhaus, B. J. Der BISA-test: ermittlung der formenkenntnis von schülern am beispiel einheimischer vogelarten. *ZfDN* **24**, 235–249. 10.1007/s40573-018-0086-7 (2018).

[51] Lückmann, K. & Menzel, S. Herbs versus trees: Influences on teenagers’ knowledge of plant species. *J. Biol. Educ.* **48**, 80–90. 10.1080/00219266.2013.837404 (2014).

[52] Lindemann-Matthies, P., Remmele, M. & Yli-Panula, E. Professional competence of student teachers to implement species identification in schools – a case study from Germany. *CEPSj* **7**, 29–48. 10.26529/cepsj.12 (2017).

[53] Tunnicliffe, S. D. & Reiss, M. J. Building a model of the environment: How do children see animals? *J. Biol. Educ.* **33**, 142–148. 10.1080/00219266.1999.9655654 (1999).

[54] Nisbet, E. K., Zelenski, J. M. & Murphy, S. A. The nature relatedness scale. *Environ. Behav.* **41**, 715–740. 10.1177/0013916508318748 (2009).

[55] Sherratt, T. N., Wilkinson, D. M. & Bain, R. S. Explaining Dioscorides’ double difference: Why are some mushrooms poisonous, and do they signal their unprofitability? *Am. Nat.* **166**, 767–775. 10.1086/497399 (2005). 16475091 10.1086/497399

[56] Gatt, S., Tunnicliffe, S. D., Borg, K. & Lautier, K. Young Maltese children’s ideas about plants. *J. Biol. Educ.* **41**, 117–122. 10.1080/00219266.2007.9656080 (2007).

[57] Prokop, P. & Fančovičová, J. The effect of hands-on activities on children’s knowledge and disgust for animals. *J. Biol. Educ.* **51**, 305–314. 10.1080/00219266.2016.1217910 (2017).

[58] Kim, H. et al. Understanding the role of biodiversity in the climate, food, water, energy, transport and health nexus in Europe. *Sci. Total Environ.* **925**, 171692. 10.1016/j.scitotenv.2024.171692 (2024). 38485013 10.1016/j.scitotenv.2024.171692

[59] Schultz, P. W. Inclusion with Nature: The Psychology Of Human-Nature Relations. In *Psychology of Sustainable Development*, edited by P. Schmuck & P. W. SchultzSpringer, Boston, pp. 61–78. (2002).

